# Context, emotion, and the strategic pursuit of goals: interactions among multiple brain systems controlling motivated behavior

**DOI:** 10.3389/fnbeh.2012.00050

**Published:** 2012-08-03

**Authors:** Aaron J. Gruber, Robert J. McDonald

**Affiliations:** Canadian Centre for Behavioural Neuroscience, Department of Neuroscience, University of Lethbridge, LethbridgeAB, Canada

**Keywords:** amygdala, dopamine, emotion, hippocampus, inhibition, Pavlovian-instrumental transfer, reinforcement learning, striatum

## Abstract

Motivated behavior exhibits properties that change with experience and partially dissociate among a number of brain structures. Here, we review evidence from rodent experiments demonstrating that multiple brain systems acquire information in parallel and either cooperate or compete for behavioral control. We propose a conceptual model of systems interaction wherein a ventral emotional memory network involving ventral striatum (VS), amygdala, ventral hippocampus, and ventromedial prefrontal cortex triages behavioral responding to stimuli according to their associated affective outcomes. This system engages autonomic and postural responding (avoiding, ignoring, approaching) in accordance with associated stimulus valence (negative, neutral, positive), but does not engage particular operant responses. Rather, this emotional system suppresses or invigorates actions that are selected through competition between goal-directed control involving dorsomedial striatum (DMS) and habitual control involving dorsolateral striatum (DLS). The hippocampus provides contextual specificity to the emotional system, and provides an information rich input to the goal-directed system for navigation and discriminations involving ambiguous contexts, complex sensory configurations, or temporal ordering. The rapid acquisition and high capacity for episodic associations in the emotional system may unburden the more complex goal-directed system and reduce interference in the habit system from processing contingencies of neutral stimuli. Interactions among these systems likely involve inhibitory mechanisms and neuromodulation in the striatum to form a dominant response strategy. Innate traits, training methods, and task demands contribute to the nature of these interactions, which can include incidental learning in non-dominant systems. Addition of these features to reinforcement learning models of decision-making may better align theoretical predictions with behavioral and neural correlates in animals.

## Introduction

Natural environments pose numerous challenges to animals seeking to survive and reproduce. Advantage is gained by adapting behavior so as to exploit new opportunities and avoid hazards. The study of these adaptations has enjoyed a rich and active history. Pioneering animal learning psychologists of the mid-twentieth century were divided among those who viewed behavior as the learning of stimulus–response habit associations driven by reinforcement (Thorndike, 1911; Hull, 1943) and those who postulated that animals used internal representations of environmental contingencies in order to select actions achieving desirable goals (Tolman, 1932). Habitual responses can be generated quickly and accurately with simple learning schemes, but are slow to change in the face of changing environmental contingencies between antecedents (e.g., stimuli, events, actions) and outcomes. Conversely, goal-oriented responses can adapt quickly, but involve more complex learning and control schemes explicitly encoding goal values and contingency-dependent strategies. It is now generally accepted that multiple forms of learning, including both habit and goal-oriented systems, are distributed among multiple brain structures and interact so as to control actions in rodents and primates (McClelland et al., 1995; Balleine and Dickinson, 1998; Wise and Murray, 2000; Cardinal et al., 2002a; White and McDonald, 2002; Doya, 2008).

**Figure 1:**
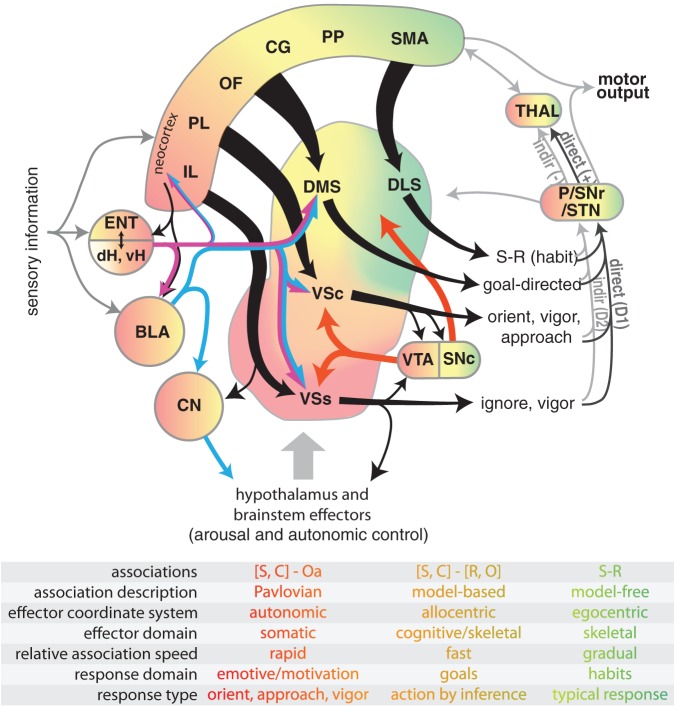
**Diagram illustrating select connectivity of brain structures involved in the voluntary control of rodent behavior.** Four regions of rodent striatum are indicated by labels and color; the color gradient approximates the gradient of afferent projections (Voorn et al., 2004). Corresponding color in the other structures represents general projection topography. Note that ventral hippocampus has direct projections outside of the hippocampal formation, whereas the dorsal hippocampus does not (see text). Tapered arrows indicate highly convergent input. Output of the striatum can proceed through two distinct pathways to reach the thalamus and other targets: a “direct” pathway that has a disinhibitory effect (+), and an indirect pathway that has an inhibitory effect (−). These pathways are innervated by separate populations of medium spiny neurons in the striatum that predominantly express D1 (direct) or D2 (indirect) dopamine receptors. Projections from dopamine neurons in the VTA and SNc are shown in red. The overarching organization is that of loops linking neocortex, basal ganglia, and thalamus. The table indicates some characteristic features of dissociated behavioral control systems, with color indicating corresponding brain structures. Abbreviations for the table: stimulus (S), context (C), affective outcome (Oa), response (R), specific outcome (O). Abbreviations for the main figure: DLS, dorsolateral striatum; DMS, dorsomedial striatum; VSc, core of the nucleus accumbens in ventral striatum; VSs, shell of the nucleus accumbens in ventral striatum; VTA, ventral tegmental area; SNc, substantia nigra pars compacta, SNr, substantia nigra pars reticulata, P, pallidum; STN, subthalamic nucleus; dH, dorsal hippocampus; vH, ventral hippocampus; ENT, entorhinal cortex; BLA, basolateral nucleus of the amygdala; CN, central nucleus of the amygdala. The following regions of neocortex are labeled: IL, infralimbic; PL, prelimbic; OF, orbitofrontal; CG, cingulate, PP, parietal; SMA, sensorimotor.

Here, we review key literature regarding the behavioral significance of processing in and among rodent frontal cortex, striatum, amygdala, hippocampus, hypothalamus, and brainstem modulatory systems. In addition to the formation of segregated circuits among these structures for mediating (i) habits involving dorsolateral striatum (DLS) and (ii) goal-directed control involving dorsomedial striatum (DMS), they also form (iii) an emotional memory system involving the ventral striatum (VS) and its limbic inputs that exerts an important influence on behavior (Figure 1). This emotional system engages postures, attention, and autonomic responses rather than selecting specific actions, but these are nonetheless important for engaging and invigorating operant responses as well as influencing behavioral flexibility. We propose this emotional system serves to contextually gate responses to stimuli based on associated valence, and furthermore the gating out of neutral stimuli and suppressing unrewarding responses is an important feature for behavioral control. When a stimulus passes the triage threshold of this system, operant responding is then determined by a competition between a habit system and a goal-directed system that is sensitive to specific outcomes and complex task demands. The ventral emotional triage system has a high capacity, forms memories rapidly, and forgets associations slowly such that stimuli associated with rewards engage attention and responding, which may allow the slower-learning goal and habit systems to solve tasks efficiently. In an attempt to synthesize a coherent framework cutting across the large and complex rodent literature on these brain systems, we first review key evidence for functional significance of structures individually before reviewing evidence of their interactions.

## Amygdala: emotional learning and memory system

Research on the functional significance of the amygdala has consistently implicated this brain structure in emotional learning and memory (Kluver and Bucy, 1939; Weiskrantz, 1956; Goddard, 1964; Bagshaw and Benzies, 1968). Several studies in humans have demonstrated a reliable correlation between amygdala activation and emotionally valenced stimuli (Breiter et al., 1996; Reiman et al., 1997), which can even occur without subjects' awareness (Whalen et al., 1998). Experiments with animals has likewise revealed an important role for the amygdala in rapidly forming associations between environmental stimuli and positive or negative affective events in a process called classical (or Pavlovian) conditioning (Pavlov, 1927). The representations formed by these associations are essential for many behaviors, as they can be used to initiate general approach or avoidance behaviors as well as modulate overt responses via interactions with instrumental memory systems (Davis et al., 1982; Cador et al., 1989; Everitt et al., 1989; Hiroi and White, 1991b).

The critical involvement of the amygdala in emotional learning and memory processes is supported by the unique and extensive reciprocal connectivity of this structure to an interesting array of cortical and subcortical targets (White and McDonald, 2002; Ledoux, 2007). Briefly, the amygdala receives extensive sensory input from the thalamus or cortex from all of the major sensory modalities. This feature of amygdala connectivity suggests that it has online access to multimodal information about the external environment. The amygdala also has extensive reciprocal connections with portions of the hypothalamus, brainstem, hippocampus, and VS (Petrovich et al., 2001). These brain areas control many autonomic functions like heart rate, respiration, hormone release, and neurotransmitter release that occur during both negative and positive experiences (Kapp et al., 1990; Davis, 1992b; White and McDonald, 2002; Everitt et al., 2003). This feature of amygdala anatomy provides the system with information about negative and positive affective states, as well as a pathway to influence physiological responses accordingly. Mechanisms of synaptic plasticity in the amygdala allow for rapid association among convergent sensory and affective information for later use (Maren and Quirk, 2004). Accordingly, neurons in the amygdala respond to sensory stimuli from various modalities that are paired with positive or negative states, but responses rapidly habituate if not paired with biologically significant cues (Ben-Ari and Le Gal La Salle, 1974; Schoenbaum et al., 1998).

The amygdala is anatomically divided into multiple nuclei with differing connectivity and functional specialization. The basolateral nucleus is defined by extensive reciprocal connectivity with the thalamus, cortex, and VS (Veening, 1978; Simon et al., 1979). Numerous research groups have provided evidence implicating the basolateral nucleus in the acquisition, storage, and retrieval of stimulus-reward associations (Schwartzbaum, 1960; Spiegler and Mishkin, 1981; Cador et al., 1989; Everitt et al., 1991; Kentridge et al., 1991; McDonald and White, 1993). An example of this kind of learning is a conditioned cue preference task developed for the 8-arm radial maze. Training on this task consists of pairing a highly palatable food (sweetened cereal) with a lit arm and the absence of food with a darkened arm. During testing in the absence of food, normal rats spend more time in the previously paired arm than the unpaired arm. Importantly, rats are never trained (instrumentally) to make any voluntary movements to obtain the food reward. Rather, they simply consume the food in the presence of a specific cue. This is a straightforward demonstration of classical conditioning between sensory events and affective outcomes whereby rats learn that the light signals the presence of food and this association elicits a general approach response and thereby maintains the subjects' contact with that stimulus. Importantly, rats with damage to the lateral or basolateral nucleus of the amygdala are impaired on the acquisition of this task (McDonald and White, 1993).

These stimulus-outcome (S-O) associations can be used as conditioned reinforcers for learning instrumental responses in the absence of primary rewards (Everitt et al., 1989). In these paradigms, initial training consists of pairing a reward (e.g., access to a sexual partner) with a punctate stimulus. Following this S-O learning, these rats can then learn to respond on a lever to activate the stimulus even if the primary reinforcement is not given. Rats given neurotoxic damage to the basolateral amygdala after S-O learning do not learn a new instrumental response using the conditioned reinforcer, indicating that this structure is needed for representing valence associated with conditioned stimuli to enable additional learning. These S-O associations can also be acquired during cued instrumental training, although they do not seem to be necessary for accurate performance of the task. For example, acquisition of tasks requiring rats to generate turning responses into lit maze arms for food rewards are sensitive to lesions of DLS rather than amygdala (McDonald and White, 1993). However, the amygdala does incidentally acquire information about the signaling value of the cue in these conditions (light = reward), which can be revealed with a preference transfer test independent of the reinforced instrumental response (McDonald et al., 2004).

The lateral, basolateral, and central amygdala have also been implicated in cued aversive classical conditioning (Bagshaw and Benzies, 1968; Kapp et al., 1979; Davis, 1986; Ledoux et al., 1990; Kim et al., 1993; Antoniadis and McDonald, 2000). An example of this conditioned fear learning comes from Kapp and colleagues (Kapp et al., 1979), who utilized a classical conditioning paradigm in rabbits in which one stimulus (tone) predicted an aversive eye-shock and another stimulus (light) did not. This is another task in which animals were never trained to make voluntary movements during conditioning. They experienced the aversive event in the presence of particular cues and rapidly (within a few repetitions) learned that the tone signaled the aversive eye-shock. This association lead to an internal fear state that elicits general avoidance and involuntary effects such as lowering of heart rate (bradycardia). After sufficient training, the rabbits showed conditioned bradycardia to the tone and little or no changes in heart rate when the light was presented. Lesions to the central (Kapp et al., 1979) or basolateral (Ledoux et al., 1990) nucleus of the amygdala impairs this form of classical conditioning. Furthermore, neural activity in the central amygdala is elevated in the presence of the shock-paired stimulus but not the unpaired stimulus (Kapp et al., 1979), consistent with proposals that the amygdala signals the affective significance of stimuli associated with aversive as well as appetitive outcomes (Morrison and Salzman, 2010). The central nucleus of the amygdala is defined by extensive reciprocal connections with the brain stem and hypothalamus that allow this system to activate defensive responses following learning (Maren, 2001; Viviani et al., 2011). Note that this system is distinct from the brain structures primarily involved in generating operant responses, such as motor cortex and DLS (McDonald and White, 1993; Hikosaka, 1998). Although numerous studies have now shown that amygdala is sufficient for rapid development of fear responses evoked by discrete cues, rapid learning of fear induced by environmental context additionally involves dorsal hippocampus (Sutherland and McDonald, 1990; Kim and Fanselow, 1992). Such dissociation of cue and context learning is important, and will be discussed in more detail later.

The central nucleus of the amygdala has also been implicated in innate postural responses supporting appetitive learning. Holland and Gallagher (Gallagher et al., 1990) utilized a set of unconditioned responses by rats to novel visual (rearing) and auditory (startle) cues; these are sometimes referred to as orienting responses. These responses are maintained if cues are associated with reinforcement, but normally habituate in the absence of reinforcement (Holland, 1977). Rats with damage to the central, but not basolateral, nucleus of the amygdala show normal unconditioned orienting responses to these cues, but do not maintain these behaviors when the cues are associated with food availability (McDannald et al., 2005). Orienting responses are also altered following damage to the central nucleus of the amygdala in a Pavlovian autoshaping procedure in which rats approach food-related stimuli (Parkinson et al., 2000a). Thus, the central nucleus is involved in controlling postural responses that keep animals in contact with behaviorally relevant stimuli. Consistent with this role in guiding behavioral focus to relevant stimuli are demonstrations that amygdala is involved in attention processes after task acquisition (Gallagher et al., 1990) and signaling surprising events (Roesch et al., 2012). Some of these effects on attention and task engagement may involve amygdala innervation of hypothalamus (Petrovich et al., 2001), an integrative structure involved in arousal, autonomic control, sleep, reproduction, food intake, and other functions (Saper, 2003). For instance, hypothalamic neurons can release neurotransmitters including histamine and hypocretin that promote arousal onto cortico-limbic targets such as medial prefrontal cortex, amygdala, VS, hippocampus, and brainstem monoaminergic neurons (Parmentier et al., 2002; Takahashi et al., 2006; Haas et al., 2008; Berridge et al., 2010). Many of these target structures in turn innervate hypothalamus (Risold and Swanson, 1996; Saper, 2003; Haas et al., 2008), thus forming an interconnected network that can promote arousal and consumption (Kelley et al., 2005), or fearful responses under stressful conditions (Petrovich et al., 2001; Herman et al., 2005). The data summarized so far indicate that the amygdala rapidly associates multimodal sensory and affective signals so as to trigger freezing or orienting responses, modulate visceral function, and influence overt operant responses during instrumental learning, largely mediated via different pathways. As discussed later, amygdala input to the VS is important for learning and modulating operant responses. Thus, the associations between stimuli and affective outcomes (S-O) formed in the amygdala impact a number of brain systems involved in both early and late phases of adaptive behavioral control.

## Hippocampal formation: space, context, and disambiguation

The hippocampus and related structures are thought to rapidly acquire and store relational information about spatial, contextual, and multimodal sensory elements of episodic experiences (O'Keefe and Nadel, 1978; Sutherland et al., 1989; Maren et al., 1997; Tulving and Markowitsch, 1998). The representation of this information can be recalled at a later time to activate a variety of effectors including those involved in producing internal states, general approach, avoidance, freezing, and complex navigational behaviors in animals (White and McDonald, 2002). The anatomy of the hippocampus and its neural correlates of behavior are consistent with this view (O'Keefe and Dostrovsky, 1971; McNaughton et al., 1983; Amaral and Witter, 1989; Muller, 1996; Derdikman and Moser, 2010). The hippocampus receives extensive sensory input from all cortical sensory association areas via connections with the perirhinal and entorhinal cortices (Amaral and Witter, 1995; Lavenex and Amaral, 2000). This information appears to be processed in a distributed manner throughout the septal and temporal extent of the structure such that encoding is sparse and unique for a given input configuration (Muller and Kubie, 1987; O'Keefe and Speakman, 1987). The output of this representation may serve as an index of an episode to facilitate reactivation of activity related to the experience in other brain regions (Schwindel and McNaughton, 2011).

The classic demonstration of spatial context encoding in hippocampus comes from neurons showing “place fields” in which activation increases in specific locations in an environment. Intriguingly, place fields are unique to specific environments. The population of place cells are globally remapped to orthogonal representations (sets of active neurons with few common members) when animals are moved among different testing environments (Muller and Kubie, 1987; Leutgeb et al., 2005; Jezek et al., 2011). Within the same environment, firing rates of place cells in the place field are sensitive to factors such as head direction, idiothetic movement metrics, and cues in an environment (McNaughton et al., 1983; Muller and Kubie, 1987; O'Keefe and Speakman, 1987; Leutgeb et al., 2005). These place cells are thought to be a substrate through which a representation of the topographical relationships amongst cues in an environment are formed and stored (O'Keefe and Nadel, 1978).

Consistent with the abundant electrophysiological data implicating hippocampus in spatial information processing, rats with damage to the hippocampus are impaired on a variety of spatial learning and memory tasks. These include the standard hidden platform version of the water maze (Morris et al., 1982; Sutherland et al., 1982), 8-arm radial maze (Olton et al., 1978; Harley, 1979), and spatial discriminations (O'Keefe et al., 1975; Rasmussen et al., 1989). In addition to navigation, hippocampal dysfunction impairs rats' ability to learn about experiences in specific spatial contexts (Sutherland and McDonald, 1990; Selden et al., 1991; Kim and Fanselow, 1992; Antoniadis and McDonald, 2000). Beyond purely spatial contexts, hippocampal damage also impairs rats' ability to discriminate based on configurations of stimuli (Rudy and Sutherland, 1989; McDonald et al., 1997).

The pattern of lesion effects reported in this classic work makes it tempting to suggest that hippocampus is critical for spatial, contextual, and relational/configural associations. However, these tasks are not sufficient to fully capture the unique representational contributions of the mammalian hippocampus to behavior. An emerging body of work now suggests that many tasks can be altered in ways that make them highly sensitive or insensitive to hippocampal dysfunction. One task feature that necessitates hippocampal involvement is high cue ambiguity. For example, rats with hippocampal damage can solve binary spatial discriminations for distal but not proximal reward zones (McDonald and White, 1995a; Gilbert et al., 1998). Conversely, normal rats can solve both problems regardless of spatial ambiguity. We have shown similar effects by varying ambiguity in versions of configural association tasks (McDonald et al., 1997) and fear conditioning to context (Frankland et al., 1998; Antoniadis and McDonald, 2000). The unique hippocampal contribution to disambiguation is sometimes referred to as pattern separation (Sutherland et al., 1989; O'Reilly and Rudy, 2001), and is consistent with the high sensitivity of hippocampal neural activity to environmental factors. Interestingly, Fanselow (Fanselow, 2000) suggested that rats must form a gestalt of a test chamber by exploring it over the course of minutes before contextual fear can be acquired. This is similar to the amount of time needed for rats to form a stable hippocampal firing field in a novel chamber (Bostock et al., 1991). Recent work with genetically modified mice has shown a causal link between these by demonstrating that selective optogenetic stimulation of dorsal hippocampal neurons that encode a fear-associated place can induce freezing responses when animals are in a benign environment (Liu et al., 2012). Thus, hippocampal activity patterns are sufficient for mice to engage behaviors associated with the encoded place or episode.

In addition to ambiguity, a second task factor that appears to necessitate hippocampal involvement is temporal ordering among events. Hippocampal damage impairs the ability of rats to use previously learned sequential ordering of odor cues to make discriminations, but spares recognition of the same odors (Fortin et al., 2002). The hippocampus is also involved in tasks with delays between events. This has been shown in non-match-to-sample tasks in monkeys, wherein hippocampal damage impairs responding when delays are introduced between sample and match phases (Mishkin and Manning, 1978). A similar interaction between lesion and delay has been found for dorsal (but not ventral) hippocampal lesions in rats on a spatial delayed alternation task (Hock and Bunsey, 1998). Delays also recruit hippocampal involvement in non-spatial tasks such as trace fear paradigms, where a delay occurs between the conditioned stimulus and eyeblink response in rabbits (Kim et al., 1995) or freezing response in rats (McEchron et al., 1998). Neural encoding in dorsal hippocampus contains information about temporal order. For instance, place cells in this region activate in sequence as rats passes through their respective place fields while navigating an environment during a task. These neurons briefly reactivate in the same sequence when the animal is resting (Skaggs and McNaughton, 1996). Intriguingly, sequences coding for potential future paths are briefly generated when rats approach a choice point in a spatial task (Wood et al., 2000; Shapiro et al., 2006; Johnson and Redish, 2007; Ferbinteanu et al., 2011), suggesting that the hippocampus may be sending out a predictive signal based on past episodes of spatial trajectories. Indeed, similar reactivations have also been detected in medial prefrontal cortex (Euston et al., 2007; Peyrache et al., 2009) and VS (Lansink et al., 2009; Van Der Meer and Redish, 2009), two structures that receive prominent hippocampal input and synchronize with hippocampus (Goto and O'Donnell, 2001; Jones and Wilson, 2005; Cenquizca and Swanson, 2007; Gruber et al., 2009a; Lansink et al., 2009; Benchenane et al., 2010; Hyman et al., 2010). In addition to representing sequential order through sequential firing patterns of different neurons, temporal information of events is also encoded by the timing of action potential firing of individual neurons in the hippocampus with respect to the phase of prevalent theta-frequency oscillations of field potentials in this structure (Buzsaki, 2005; Hasselmo and Eichenbaum, 2005). These features allow for compression of temporal information into single theta cycles, and indicate that the output of the hippocampus is rich in temporal (spike phase and ordering) as well as non-temporal (which neurons activate) information.

The output projections of the hippocampus vary along the septo-temporal axis (Cenquizca and Swanson, 2007), so it is unsurprising that some function appears to vary over this axis as well (Fanselow and Dong, 2010; Bast, 2011). The dorsal (septal) hippocampus primarily projects to extrahippocampal structures in the temporal lobe such as subiculum and entorhinal cortex that in turn project to most neocortical areas (Lavenex and Amaral, 2000; Cenquizca and Swanson, 2007). A dominant dorsal hippocampal/neocortical projection is to both the anterior and posterior cingulate cortices. The projection to the posterior cingulate cortex is of particular interest because this brain area also receives strong projections from posterior parietal cortex, which has been implicated in online visual guidance of behavior (Sutherland et al., 1988). It is possible that posterior cingulate allows animals to use spatial memories to navigate via interactions between hippocampus and neocortical regions like the posterior parietal cortex. The intermediate and ventral (temporal) portions of the hippocampus project to these structures as well as to ventral medial prefrontal cortex, VS, and amygdala (Voorn et al., 2004). Although less is known about the neural signaling in these more ventral portions, they are important for translating hippocampal information into actions (Bast et al., 2009).

Both the dorsal and intermediate regions of the hippocampus are thought to be necessary for accurate spatial navigation in the water task (Moser et al., 1993; Ferbinteanu et al., 2003; Bast et al., 2009), while the ventral pole (i.e., the most ventral third of the hippocampus) may not be required. The bulk of current evidence suggests that the dorsal region is more efficient in encoding spatial information and is necessary for spatial navigation, but recent work suggests that the intermediate zone is critical for translating spatial information into action, particularly in paradigms requiring rapid learning (Bast et al., 2009). On the other hand, damage to the ventral hippocampus produces behavioral impairments in non-spatial tasks similar to damage of its forebrain targets in some cases. One example of function that varies along the septo-temporal axis and impacts forebrain targets is fear conditioning in rats. Whereas the dorsal hippocampus is involved in fear conditioning to context, the ventral hippocampus appears to be involved in fear conditioning to both context and explicit cues such as tones (Maren, 1999; Bast et al., 2001; Zhang et al., 2001). Fear conditioning can be acquired without an intact hippocampus following repeated training (Wiltgen et al., 2006; Sparks et al., 2011) through a mechanism thought to involve the amygdala (Biedenkapp and Rudy, 2009). Thus, hippocampal output to other structures needed for fear conditioning, particularly the amygdala, appears to support rapid associative learning in at least some non-spatial domains. Another behavior exemplifying functional overlap between ventral hippocampus and target regions is prepulse inhibition of the startle reflex. This is a sensorimotor process in which an acoustic startle reflex is reduced when startling stimuli are preceded by a weak prepulse stimulus, and is impaired by manipulations to ventral hippocampus, VS, or basolateral amygdala among other limbic and brainstem structures (Wan et al., 1996; Wan and Swerdlow, 1996; Koch and Schnitzler, 1997; Wan and Swerdlow, 1997). Although the ventral hippocampus does not directly mediate prepulse inhibition, it is able to modulate this phenomenon (Koch and Schnitzler, 1997; Bast and Feldon, 2003).

Ventral hippocampus and its associated medial temporal lobe structures also functionally interact with VS in learning phenomena like latent inhibition and conditioned inhibition. These are similar types of learning wherein rats rapidly cease orienting toward, or responding to, stimuli that have never been associated with reinforcement (Lubow, 1989), and both appear to be context-specific (Honey and Hall, 1989; McDonald et al., 2001). Latent inhibition is a phenomenon whereby non-reinforced stimulus pre-exposure leads to retardation of the development of subsequent conditioned responses when the stimulus is later paired with reinforcement. Latent inhibition is not disrupted by selective lesions of hippocampus *per se* (Honey and Good, 1993; Reilly et al., 1993), but is disrupted by damage to its nearby cortical target, the entorhinal cortex, or VS (Weiner et al., 1996; Coutureau et al., 1999). However, neurotoxic lesions or temporary inactivation of ventral hippocampus do disrupt the usual context-specificity of latent inhibition (Honey and Good, 1993; Maren and Holt, 2000). In *conditioned* inhibition, rats are trained to discriminate between a reinforced cue and a non-reinforced cue, resulting in accrual of excitatory conditioning to the reinforced stimulus and conditioned inhibition to the non-reinforced stimulus. The contextual specificity of conditioned inhibition is disrupted by damage to ventral hippocampus (McDonald et al., 2001, 2006). Thus, hippocampus modulates the suppression of responding to irrelevant cues in a context-dependent manner.

The data reviewed in this section indicate that the hippocampus is involved in the rapid formation (Wiltgen et al., 2006; Bast et al., 2009) and recall of associations among places, contexts, and sensory configurations, and also includes temporal elements. This processing affects many types of behaviors including spatial navigation, operant responding for rewards, fearful response, and various forms of response inhibition. These features support the conclusion by Wise and Murray (Wise and Murray, 2000) that the primate hippocampus is a critical part of a network with frontal cortex and the basal ganglia that is required for learning to generate arbitrary and flexible associations between antecedents and outcomes. This is likely true for rodents as well. Hirsh (Hirsh, 1974) noted that actions in rodents appeared to be a matter of habit in the absence of the hippocampus. This structure seems to be particularly critical when outcomes are delayed or context is ambiguous. We later discuss conditions in which this structure cooperates or competes with other brain structures for contextual control of habitual and adaptive behavior.

## Striatum: a nexus among limbic structures and parallel circuits linking cortex and basal ganglia

Two influential anatomical reviews paved the way for modern conceptualizations of functional heterogeneity in the striatum. Alexander et al. (1986) proposed five parallel circuits in the monkey connecting different portions of the cortex, striatum, pallidum, substantia nigra, and thalamus in partially closed basal ganglia-thalamocortical loops. These parallel circuits included a motor circuit, multiple prefrontal circuits, and multiple limbic circuits that were centered on the dorsal and VS. Groenewegen et al. (1990) identified similar circuits in rat brain, and an important review by McGeorge and Faull (1989) pointed to clear anatomical distinctions between the dorsolateral and DMS in the rat, with the former receiving extensive convergent projections from motor and sensory cortices and the latter defined by connectivity with prefrontal and limbic projections from hippocampus and amygdala. Subsequent anatomical data has indicated that these circuits are not independent from one another (Joel and Weiner, 1994). For instance, cortico-striatal projections can innervate large volumes of striatum that cross functional territories, providing a mechanism for cross-talk among circuits (Levesque and Parent, 1998; Zheng and Wilson, 2002; Hoover and Vertes, 2011). Furthermore, Haber et al. (2000) have suggested that the overarching organization of these circuits forms a spiral wherein the limbic circuit affects the cognitive and motor circuits. Although it is convenient to identify discrete regions of striatum, cytoarchitectural composition, and afferent innervation vary according to a dorsolateral to ventromedial gradient within striatum, rather than forming discrete boundaries between these regions (Voorn et al., 2004).

Here, we consider three functional territories in our analysis of rodent striatal function: (1) a dorsolateral “motor” sector involved in skilled movements and habits, (2) a dorsomedial “cognitive” sector involved in allocentric navigation and flexible responding for strategic acquisition of goals, and (3) a ventral “limbic” sector incorporating the core and shell of the nucleus accumbens in the VS that are involved in approach behaviors, arousal, extinction, and response vigor. We first briefly review key literature on the function of each sector before turning to their interaction with each other and other cortico-limbic structures.

### Dorsolateral striatum (DLS): stimulus-response habits

The rodent DLS appears to primarily bring reinforcement-related operant movements under specific stimulus and temporal control as a result of repeated reinforced stimulus-response (S-R) experiences, which can eventually form habits (Devan et al., 2011). These movements are usually more complex than postural or orienting responses mediated by brainstem structures (Whishaw et al., 2004). Habit formation can be mediated by associative conditioning specific to the reinforced cue (McDonald et al., 2001). This is supported by neural recordings in rat DLS showing responses selective for task-related cues and sensory-motor processing (Gardiner and Kitai, 1992; White and Rebec, 1993).

Consistent with this view, rats with neurotoxic lesions of the DLS are impaired in various types of simple discrimination tasks using a variety of cues and reinforced responses (Packard et al., 1989; Reading et al., 1991; McDonald and White, 1993; McDonald and Hong, 2004). In such studies, instrumental responses (e.g., lever press or egocentric turns) must be generated in response to a stimulus to receive food reinforcement. One example is a task in which rats must push a lever if a light was present, or pull a chain if a tone was present. This task requires acquisition of S-R associations for optimal performance and cannot be solved by instrumental (response-outcome; R-O) or Pavlovian S-O associations. Rats with neurotoxic lesions of the DLS are impaired in the acquisition and retention of this task, even if the number of reinforcers is equated across groups or the motoric requirements are reduced (Featherstone and McDonald, 2004).

Further evidence that the DLS is involved in S-R learning comes from a set of studies using non-discriminative instrumental conditioning procedures (Yin et al., 2004). Briefly, rats with DLS lesions were trained to lever press for sucrose reward. Following training, the reward was devalued by injecting subjects with a substance that induced a conditioned taste aversion. Normal rats reduce responding to the lever associated with sucrose availability following this devaluation, and rats with damage to the DLS also show this effect. In the final stage of testing, both groups are returned to the operant chambers and given an extinction test in which the lever was available but no sucrose was delivered. Normal rats responded on the lever despite the fact that they recently reduced sucrose consumption, while rats with DLS damage did not respond despite also showing the devaluation effect. Temporary inactivation of the DLS produces similar effects (Yin et al., 2006), suggesting that S-R representations encoded and stored in the DLS are insensitive to outcome devaluations in instrumental learning situations. Thus, when DLS is damaged, control of behavior is mediated by other brain regions such as the DMS that are sensitive to outcome devaluations.

### Dorsomedial striatum (DMS): space and flexible responses

Accumulating evidence implicates the DMS in a range of cognitive processes including behavioral flexibility, allocentric navigation, and instrumental learning (Devan et al., 2011). This view is consistent with the connectivity of the DMS. This brain region receives glutamatergic input from the entorhinal cortex, subiculum, hippocampus, amygdala, thalamus, piriform, and prefrontal cortices (McGeorge and Faull, 1989; Voorn et al., 2004). The anatomical links between the hippocampal formation and DMS are complex. First, the subiculum projects to the most medial portions of the striatum (Groenewegen et al., 1987). Second, hippocampal output layers of the entorhinal cortex also project to the DMS (Krayniak et al., 1981; Swanson and Kohler, 1986). Third, the posterior cingulate cortex receives indirect input from the dorsal hippocampus and sends a strong projection to the DMS (McGeorge and Faull, 1989), which might provide a unique hippocampal/posterior cingulate representation to influence complex navigational abilities using visual information (Sutherland et al., 1988). Finally, the ventral hippocampus projects to portions of the medial prefrontal cortex which then project to the DMS (McGeorge and Faull, 1989). Interestingly, damage to any of the indirect sources of hippocampal input to the DMS results in impairments in place learning in the water task (Schenk and Morris, 1985; Sutherland et al., 1988; Kolb et al., 1994; Ferbinteanu et al., 1999). It is thus not presently possible to determine if any region of hippocampus has preferential influence on DMS.

DMS output targets nuclei and thalamic regions that can influence voluntary behavior (Gerfen, 1992). Furthermore, neural correlates in rodent DMS have been linked to various aspects of spatial navigational behaviors including neurons that show direction, location, and movement selectivity (Wiener, 1993; Kim et al., 2009; Mizumori et al., 2009). The location-specific cells are similar to those found in hippocampus except that the DMS “place cells” are of lower resolution. These data suggest that the DMS can utilize input from hippocampus and associated structures in some situations. Given the prominent representation of space in the hippocampus, it is thus not surprising that DMS damage impairs behaviors that require the flexible use of spatial navigation. One example comes from a variant of the water maze in which rats can swim to a submerged platform to escape the water (McDonald et al., 2008a). If the platform location is consistently moved every eight trials, normal rats will eventually learn to navigate toward the platform on the second trial after a location switch. DMS lesioned rats are impaired in the task, but do show within-session learning. Conversely, rats with hippocampal damage show severe impairments with little within-session improvements (Ferbinteanu et al., 2003). This suggests that DMS damage impairs response flexibility after a switch rather than eliminating navigation abilities. Indeed, DMS lesions impair spatial reversal learning for rewards (Castane et al., 2010), which also explicitly requires flexibility in navigation. Thus, the DMS appears to be an important node for translating navigational information, likely involving intermediate hippocampus (Bast et al., 2009), into actions that are rapidly adaptable across consecutive trials.

The role of DMS in response flexibility also extends to discriminations based on non-spatial features of multimodal cues. For instance, rats with DMS damage are able to acquire discrimination based on one dimension (e.g., light or tone) of a compound stimulus, but are impaired when they have to switch dimensions for proper discrimination (Ragozzino et al., 2002). Furthermore, neurons in rodent DMS encode stimuli and actions in tasks requiring little spatial navigation (Ito and Doya, 2009; Kimchi and Laubach, 2009), further supporting a role beyond allocentric navigation.

DMS is also involved in some types of instrumental (R-O) learning (Adams and Dickinson, 1981). These experiments typically require the subject to make a response (lever press) to obtain a desired outcome (food). This form of learning is susceptible to devaluation procedures during early phases of training but not later phases. This is thought to reflect a gradual transfer of behavioral control to S-R habit systems that are insensitive to devaluation. Various experiments provide evidence that the DMS mediates R-O associations, whereas the DLS does not (Yin et al., 2004, 2005; Yin and Knowlton, 2006). Such R-O associations would allow animals to utilize expected outcomes to select responses, which is a hallmark of a flexible, goal-oriented, control system (Adams and Dickinson, 1981; Daw et al., 2005).

### Ventral striatum and related structures: gateway from emotional memory to instrumental action

The VS has been characterized as a “limbic–motor interface,” in which information about reward, context, and motivational drive is integrated to guide motivated behavior (Mogenson et al., 1980). This region receives convergent glutamatergic input from the prefrontal cortex, hippocampus, and amygdala, as well as dopaminergic input from the ventral tegmental area (Brog et al., 1993; Lynd-Balta and Haber, 1994; Wright and Groenewegen, 1995). Outputs project to brain regions associated with generation of motor behaviors (Groenewegen and Russchen, 1984; Heimer et al., 1991), as well as midbrain dopamine and hypothalamic neurons (Heimer et al., 1991; Groenewegen et al., 1993) that can modulate arousal and autonomic function (Hilton, 1982; Sutcliffe and De Lecea, 2002). Neurons in VS core fire following and in anticipation of task-related events such as cues and reinforcements, and many of these responses also encode the value of anticipated outcomes such that firing rates are higher prior to preferred reinforcements (Carelli and Deadwyler, 1994; Nicola et al., 2004; Lansink et al., 2008; Ito and Doya, 2009; Kim et al., 2009; Kimchi and Laubach, 2009; Van Der Meer and Redish, 2009; Goldstein et al., 2012). Such prevalent reward-related modulation of activity could aid animals in making economic choices by computing the relative value of future actions or states. However, many of these same recording studies show little activity predictive of choice prior to action selection, suggesting that overt actions are selected elsewhere (Ito and Doya, 2009; Kim et al., 2009; Kimchi and Laubach, 2009; Goldstein et al., 2012). Furthermore, rats with VS damage are still sensitive to changes in the value of instrumental contingency (Balleine and Killcross, 1994). This is consistent with the proposal that goal-directed control requires circuit processing involving DMS that can operate (e.g., compute value) independently from other striatal loops. Indeed, choice-related activity is more prevalent and adapts more quickly following changes in reward contingency in DMS as compared to VS (Ito and Doya, 2009; Kimchi and Laubach, 2009). If the VS is not necessary for outcome valuation and is not generating signals predictive of upcoming actions, what is the role of the prevalent value signal in the VS that precedes choices and rewards?

Like the amygdala, the VS core and its dopaminergic input appear to impart motivational effects that bring the animal in contact with task-related stimuli and invigorates operant responding (Cardinal et al., 2002a). Cues associated with rewards attract attention and elicit a conditioned response of locomotor approach in a variety of species (Brown and Jenkins, 1968; Sidman and Fletcher, 1968; Wilcove and Miller, 1974), which bring the animal in contact with the conditioned stimulus (autoshaping) or sources of reward (conditioned magazine approach) and thereby benefit learning in appetitive tasks. These phenomena are impaired in rats by VS core damage made either during or after task acquisition, suggesting that VS plays an ongoing role in behavior (Parkinson et al., 2000b; Cardinal et al., 2002b). The VS core is also involved in modulating the vigor of operant responding. VS core damage reduces rates of operant responding (Balleine and Killcross, 1994), and abolishes (Corbit and Balleine, 2011) the normally invigorating effects of non-contingent Pavlovian conditioned stimuli through a phenomenon called Pavlovian-to-instrumental transfer [PIT; (Estes, 1943; Lovibond, 1983)]. One version of PIT involves instrumental training on two levers that result in different reinforcement outcomes. Next, Pavlovian conditioning is used to associate the same outcomes with novel conditional stimuli (tone and light). During a transfer test, one of these conditioned stimuli is presented while the subjects are allowed to respond on the levers. The PIT effect is the change in response rate evoked by the conditioned stimulus, which can be specific to a particular response [one lever; (Colwill and Rescorla, 1988)] or can exert a non-specific modulation of motivation [both levers; (Dickinson and Dawson, 1987)]. PIT involves processing in a number of structures projecting to VS, including neuromodulator-releasing neurons, as described subsequently.

Inputs from dopamine-releasing neurons are an important component of VS function (Yun et al., 2004), and dopamine depletion in VS results in similar impairments as VS damage. For instance, dopamine depletion in VS core impairs the acquisition and performance of autoshaping (Parkinson et al., 2002). The behavioral significance of dopamine in the VS core has been nicely demonstrated in a recent set of experiments utilizing a detailed analysis of behavior on multiple tasks following dopamine depletion (Nicola, 2010). Nicola concluded that the primary deficit was reduced initiation of operant responding due to a reduced capacity to generate appropriate approach behaviors toward task-related apparatuses, a process he termed “flexible approach.” However, once dopamine-depleted rats engaged in a chain of operant responding, their behavior was not different from control animals. Supporting this hypothesis, VS core neurons fire in response to reward-predictive cues that trigger flexible approach (Nicola et al., 2004; Day et al., 2006; Wan and Peoples, 2006). These firing responses depend on afferents from the basolateral amygdala and prefrontal cortex (Ambroggi et al., 2008; Ishikawa et al., 2008), suggesting that Pavlovian effects of the amygdala and other structures may be exerted through the VS. The flexible approach hypothesis is consistent with the hypotheses that dopamine in the VS core is involved in assigning behavioral salience to stimuli (Berridge and Robinson, 1998) and mediating effortful responding (Salamone et al., 2009). One example of such effects is the finding that increasing VS core dopamine levels by local amphetamine injection potentiates PIT for food rewards (Wyvell and Berridge, 2000). Conversely, blocking D1 type dopamine receptors in VS core reduces responding for intracranial self-stimulation (Cheer et al., 2007). These data suggest that the VS core is involved in the engagement of an activity and modulating response vigor rather than the overt selection of actions, and this function depends on inputs from prefrontal cortex and amygdala, as well as dopamine neurons (Hauber and Sommer, 2009; Salamone et al., 2009).

Although less is known about the behavioral significance of the VS shell region, it appears to share some functional overlap with the VS core. For instance, damage to VS shell impairs the outcome-specific form of PIT in which response invigoration is specific to actions leading to the same outcome as the Pavlovian conditioned stimulus (Corbit and Balleine, 2011). Additionally, injection of amphetamine into the shell region increases lever pressing for conditioned reinforcers associated with food (Parkinson et al., 1999). These data suggest function similar to the proposal that VS core is part of a circuit for initiating responding or reinstatement of behavior in drug-taking or fear-conditioning paradigms (Peters et al., 2009). However, the VS shell appears to potentiate specific responses, whereas the VS core promotes approach and general response vigor (Parkinson et al., 1999; Corbit and Balleine, 2011). Contrasting these potentiating effects, the shell is also implicated in extinction learning (Peters et al., 2008), in which formerly positive or negative contingencies of stimuli are changed to a neutral contingency such that associated behaviors gradually extinguish. Extinction learning creates an inhibitory memory trace distinct from that created by conditioning (Rescorla, 2004), and is thus an active learning process rather than a “forgetting” of the initial associations. Extinction involves interactions among the VS shell, ventromedial prefrontal cortex and central amygdala, which have been described as a pathway for actively suppressing actions, and also for engaging avoidance/freezing behavior (Peters et al., 2009). The VS and its cortico-limbic afferents are also important for some other forms of response suppression including latent inhibition (Gal et al., 1997). Thus, the VS and associated structures are involved in processing stimuli with negative and neutral valence, as well as positive valence. Under the triage framework advocated here, the promotion or suppression of responding involving VS described in this section can be explained by mechanisms distinct from the proposed “go” and “stop” functions of the dorsal striatum that are specific for particular actions (Chevalier and Deniau, 1990; Mink, 1996). Rather than generating a suppression signal that competes with a planned action (Aron and Poldrack, 2006), the VS and associated cortico-limbic structures may instead gate sensorimotor and/or neuromodulatory processes that support the generation of motor programs thus preventing the neural substrate of actions from forming.

Many VS-dependent behaviors have a strong contextual component likely involving input from ventral hippocampus. Damage to VS or ventral hippocampus decreases contextual sensitivity of drug reinstatement, fearful responses, and latent and conditioned inhibition (Honey and Good, 1993; McDonald et al., 2006). Neural recordings further support the notion that hippocampal output shapes VS representations. VS activity shows phase precession to theta oscillations thought to depend on hippocampal input, and VS activity has a spatial component wherein cells activate during approach to reward zones (Lansink et al., 2008, 2009; Van Der Meer and Redish, 2009, 2011). Thus, the VS appears to multiplex information about space, cues, and affective outcomes, consistent with its role in developing preferences for places associated with positive valence (Hiroi and White, 1991a; White et al., 1991). Such information is expected in a system that engages approach to places (e.g., feeders) based on affective associations of stimuli. Interestingly, activity of neurons in the DMS have been reported to be more sensitive to values of specific outcomes than those in the VS (Kimchi and Laubach, 2009), suggesting that encoding of affective value may somewhat dissociate from action-specific values. This would be useful in scenarios such as reward reversal paradigms in which the affective value of a particular cue-response pair becomes negative or neutral, while others become positive. A more slowly changing affective signal related to tasks rather than particular cues or responses would be useful for keeping animals engaged in tasks so as to solve new discrimination problems, despite temporary reductions in rewarded responding.

## Interactions among limbic, cognitive, and motor circuits

The data reviewed thus far indicate that the VS, amygdala, ventral hippocampus, and ventromedial prefrontal cortex form a network involved in linking stimuli and context with affective value, and engaging appropriate autonomic and postural responses. On the other hand, the control systems involving medial or lateral dorsal striatum are involved in selecting specific actions. These structures can cooperate, compete, or interfere depending on training and task demands, revealing a dynamic interaction for the control of behavior.

### Hippocampal-amygdala interactions in place preference and contextual fear

One form of competition among neural structures appears to involve blocking access to a common output node. Such an interaction appears to occur during acquisition of conditioned place preference (CPP). CPP is an appetitive classical conditioning paradigm that uses distal spatial cues as the conditioned stimuli. Acquisition of this task normally involves a synergistic interaction between the amygdala, hippocampus, and VS (McDonald and White, 1993, 1995b). However, rats with lesions of ventral hippocampus or fornix (output fibers from hippocampus and associated structures) show *enhanced* acquisition of CPP, suggesting that the ventral hippocampal circuit normally retards control of behavior by amygdala in this task (McDonald and White, 1993, 1995b; Ferbinteanu and McDonald, 2001). This could be mediated by competition between these structures for access to the VS, which is necessary for CPP (Hiroi and White, 1991b; White et al., 1991). Pre-training exposure to the maze could be sufficient for hippocampal input to retard subsequent amygdala control of VS activity (Ferbinteanu and McDonald, 2001). Such a blockade of amygdala input to VS by hippocampal input has been reported in experiments using electrical stimulation of these afferents (Mulder et al., 1998).

Another task revealing complex interactions among these structures is fear conditioning to context. It is acquired rapidly with an intact hippocampus, but can be acquired without it following repeated training (Wiltgen et al., 2006; Sparks et al., 2011). The non-hippocampal memory is thought to involve the amygdala (Biedenkapp and Rudy, 2009), which associates some salient feature of the context with the negative event after multiple repetitions. Conversely, the hippocampus is thought to rapidly create a complex and unique representation reflecting all of the features of the context, which can be used by amygdala to facilitate context-based learning (Antoniadis and McDonald, 2000; Fanselow and Poulos, 2005). However, fear memories learned when hippocampus is inactivated can be recalled in subsequent sessions when hippocampus is again inactivated, but are not properly recalled when hippocampus is left online (Sparks et al., 2011), suggesting another example of interference or competition produced by the hippocampal output. Thus, data from CPP and fear conditioning paradigms suggest that hippocampal output may both facilitate learning to context in amygdala while also suppressing amygdala influence on VS in some instances. On the other hand, hippocampal place cells acquire responses to auditory conditioned stimuli in fear conditioning paradigms (Moita et al., 2003), suggesting that amygdala representations may also influence hippocampal processing.

### Amygdala modulation of operant response: pavlovian-instrumental transfer and emotional responses

Various experiments have shown examples of PIT in which an amygdala-based Pavlovian association supports acquisition and maintenance of an arbitrarily reinforced instrumental response (Holland and Gallagher, 2003; Zorawski and Killcross, 2003; Corbit and Balleine, 2005). The general and outcome-specific form of PIT dissociate among multiple brain structures. The general form depends on central nucleus of the amygdala (Holland and Gallagher, 2003), VS core (Hall et al., 2001), and dopamine input to VS (Wyvell and Berridge, 2000; Lex and Hauber, 2008). The outcome-specific form requires basolateral amygdala (Blundell et al., 2001; Corbit and Balleine, 2005), VS shell (Corbit et al., 2001), DLS (Corbit and Janak, 2007) and dopamine neurons (El-Amamy and Holland, 2007). This is consistent with the hypothesis that VS core circuits are involved in general invigoration, while DMS/DLS circuits are selective for actions. Interestingly, PIT is enhanced with training (Holland, 2004), suggesting that the S-R system is more susceptible to this phenomena than the goal-oriented control systems and PIT is therefore more prominent as the S-R system takes over behavioral control.

Pavlovian associations can also attenuate instrumental responding and reflex amplitude. An example is conditioned emotional responding. In this paradigm, rats receive aversive outcomes paired with a cue (light) in one room, and learn operant responding for food in another room. Animals drastically reduce responding when the Pavlovian cue is presented during the operant task, likely owing to a fearful state that suppresses instrumental responding (Estes and Skinner, 1941; Leaf and Muller, 1965). Rats with amygdala damage show normal acquisition of the instrumental response, but do not reduce responding when the signal for an aversive event is presented (Davis, 1990; Weiner et al., 1995). These fearful Pavlovian associations can also modulate reflex amplitudes such as the acoustic startle reflex (Davis, 1992a). Collectively, these data indicate that Pavlovian (S-O) associations in the amygdala can invigorate or suppress instrumental responding and reflexes, possibly by evoking neural activity (Stuber et al., 2011) or dopamine release (Howland et al., 2002) in VS. The dependence of PIT on multiple interconnected brain structures presents another example in which systems interactions are needed to support behavioral phenomena.

### Allocentric versus egocentric responding

Some learning paradigms can be acquired in parallel by multiple systems, particularly in tasks that can be solved my multiple strategies. For instance, rats trained to retrieve food in one location of a plus maze use an allocentric place strategy early in training and then switch to an egocentric stimulus-response (S-R) strategy later in training (Tolman et al., 1946). Subsequent studies showed that hippocampus was necessary for performance in early phases, while DLS was necessary for later phases in this task (Packard and McGaugh, 1996; Packard, 1999). This pattern of data suggests that DLS-dependent control gradually builds sufficient associative strength to take over from hippocampal-dependent control, yet either control strategy can solve the problem. It further suggests the possibility that the hippocampus reaches asymptotic associative strength levels faster than the DLS system, which is consistent with other studies of spatial navigation and contextual fear (Muller et al., 1987; Rudy et al., 2004; Stote and Fanselow, 2004; Wiltgen et al., 2006).

**Figure 2:**
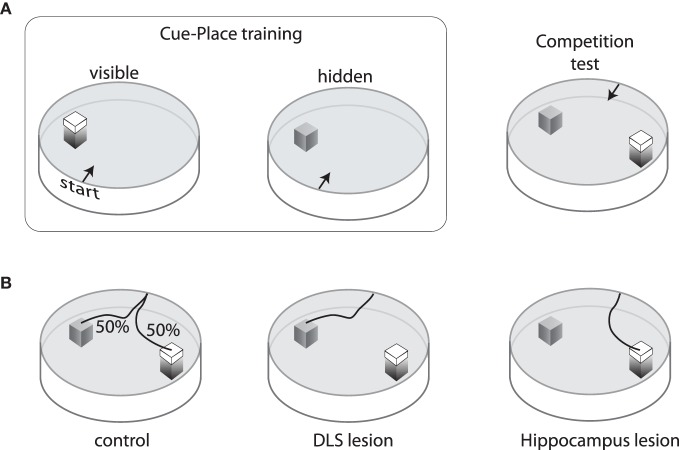
**Training and performance on the cue/place water task. (A)** Rats are trained for three days to swim from one of four start points to a visible platform located in the same spatial position relative to the pool and room cues. On the fourth day, the visible platform is removed and an invisible (submerged) platform is put in its place. This sequence is repeated three times so that each animal receives nine visible platform days of training and three invisible training days. After training, a competition test is performed in which the visible platform is moved to a new location in the pool and the invisible platform remains in the original position. Rats start from one of two points equidistant from the platforms. **(B)** On this competition test, control rats are equally likely to choose either the cue or the place response. Rats with neurotoxic damage to the hippocampus mainly swim to the visible platform, whereas those with neurotoxic damage to the dorsolateral striatum mainly swim to the invisible platform.

The DMS appears to be critical for hippocampal-based control to compete with the egocentric control mediated by DLS. An example of this (McDonald and White, 1994) was demonstrated using a modified version of the water maze task in which rats were trained to navigate to a platform that was located in the same spatial position but either visible or submerged in 12 training sessions (Figure 2A). Subjects were then given a choice between a submerged platform in the original spatial location and a visible platform in a novel location. Rats with damage to the hippocampus mainly chose the cued location (visible platform), while rats with damage to the DLS mainly chose the remembered place (submerged platform). Interestingly, the control subjects split on this competition test; about half chose the visible platform and the others chose the original spatial location (Figure 2B). Rats who chose the place response were better place learners during training, suggesting that trait differences in the relative strength of these two systems determined which gained control over behavior. Rats with DMS damage could acquire both cue-based and place-based solutions, but mainly chose the visual platform in the cue-place competition test, suggesting that the hippocampal representation could not compete for behavioral control during the test (McDonald and White, 1994; Devan et al., 1999). Subsequent cross-lesion studies have shown that interaction between DMS and hippocampus is needed for behavioral flexibility in navigation to cues and remembered places (Devan and White, 1999).

These data reveal competition between egocentric responding mediated by DLS and allocentric responding to remembered places mediated by hippocampus-DMS interactions. However, asymptotic performance on other navigation tasks that require both sensory driven responses (tactile-turning responses) and within-session spatial information (what arms have been previously visited) requires both intact DLS and hippocampus (McDonald et al., 2006). Thus, the nature of the interaction (competitive versus cooperative) between these control systems depends on task demands.

### Necessary and incidental associations acquired during visual discrimination learning: context-specific inhibition by ventral hippocampus

We have recently developed a task revealing a potentially novel subclass of associations that are acquired and stored by different neural systems, and that interact with one another in ways not previously described. These associations appear to be incidentally acquired during acquisition of a visual discrimination task, and affect response flexibility when task contingencies change. In this task, rats first learn an S-R association between a particular stimulus (e.g., light on) and response (turn) that is repeatedly reinforced in a particular context “A” (room). Responses to a CS- (e.g., light off) are not reinforced. The effects of reversal learning among these stimuli in context A or a different context B show that reversal is slower in context A, in which the original training occurred (McDonald et al., 2001). Furthermore, a transfer test in which subjects reversed in context B, and were then re-tested in context A, revealed that an inhibitory association to the CS- acquired in the original context (A) still remained despite the reversal experience in context B. Consistent with other tasks, the DLS is necessary for acquisition of the S-R association (McDonald and Hong, 2004). Superimposed on this association are several apparent incidental associations. One of these associations is Pavlovian in nature and appears to be acquired and stored in the amygdala (McDonald and Hong, 2004). The amygdala representation is not context-specific, enhances the speed of reversal learning, and can be revealed during a conditioned-cue preference transfer test. In contrast to the previously described PIT effects involving amygdala, this novel amygdala-based S-O association might extinguish quickly and reduce general approach responses toward the illuminated arms, which could accelerate reversal learning when that representation is available. Another novel association is a context-specific inhibitory association acquired by the ventral hippocampus. This representation inhibits reversal learning in the same context as original learning, but does not seem to otherwise influence task acquisition or performance (McDonald et al., 2006, 2007). This is a unique demonstration because in most instances the hippocampal representation supports engagement, rather than suppression, of context-appropriate behavior (McDonald et al., 2007).

One emergent question is whether the striatum interacts with hippocampus in this context-specific inhibitory process. To partly assess this question, rats with neurotoxic damage to the DMS were trained on the previously-described visual discrimination task. DMS damage was expected to remove the context-specific inhibitory effect by disrupting functional interaction with hippocampus, analogous to the effects of DMS damage on spatial navigation. Surprisingly, damage to the DMS resulted in *enhanced* inhibition of reversal learning in the same context (McDonald et al., 2008b). We argued that this resulted from the elimination of a DMS-frontal cortex circuit that normally suppresses a parallel circuit linking VS-ventral hippocampus and ventromedial prefrontal cortex involved in extinction processes (McDonald et al., 2007).

### Cortical interactions

Many of the behaviors thus far described also involve cortical processing. A thorough treatment is beyond the scope of this review. Briefly, the several regions comprising the rodent prefrontal cortex are implicated in numerous mnemonic, emotive, and cognitive functions supporting flexible responding to achieve goals (Dalley et al., 2004; Kesner and Churchwell, 2011). Among the many identified tasks involving subregions of prefrontal cortex, damage to the orbitofrontal region impairs outcome devaluation (Gallagher et al., 1999) and reversal learning (Schoenbaum et al., 2002), while damage to the medial prefrontal region impairs shifting discrimination among different stimulus dimensions (Joel et al., 1997; Ragozzino et al., 1999b; Birrell and Brown, 2000) as well as flexibility in place/response navigation (Ragozzino et al., 1999a). DMS receives input from these cortical regions (McGeorge and Faull, 1989), and DMS damage yields similar deficits in devaluation (Yin et al., 2005), reversal learning (Ragozzino, 2007), and flexibility in place/cue navigation (Devan et al., 1999; Ragozzino et al., 2002). Functional overlap between ventromedial prefrontal cortex and VS has been noted in acquisition and extinction of drug-taking and fearful responses (Peters et al., 2009). These data suggest that processing in cortico-striatial-pallidal/nigral-thalamic loops is sensitive to disruption at multiple points. Furthermore, the hippocampus, amygdala, and midbrain neuromodulatory neurons are reciprocally connected with prefrontal cortex as well as other nodes of the loop (Voorn et al., 2004). Such rich connectivity allows for complex interaction among these structures.

## Mechanisms of competition

### Inhibition within the striatum

One pervasive type of interaction that has emerged from the study of flexible responding is competition among control systems involving the striatum. Examples include selection of one operant response over others (Mink, 1996), allocentric versus egocentric navigation (Packard, 1999), or control over response extinction (McDonald et al., 2007). Indeed, anatomical and physiological data support the notion of inhibition among circuits flowing through striatum and other component nuclei of the basal ganglia. The majority of glutamatergic input to the striatum converges onto medium-sized spiny projection neurons (SPN), the primary neuron type in the striatum (DiFiglia et al., 1976; Chang et al., 1982). SPN receive a unique and rich set of inputs (Kincaid et al., 1998), and release the normally inhibitory transmitter GABA onto targets in other component nuclei of the basal ganglia as well as neighboring SPN (Somogyi et al., 1981; Bolam and Izzo, 1988; Tunstall et al., 2002). This latter feature has led to the development of many theories of the basal ganglia in which the striatum works as a competitive network wherein the most active SPN will suppress the activity of others through inhibitory collaterals (Groves, 1983; Wickens et al., 1995; Suri and Schultz, 1998; Gruber et al., 2006; O'Reilly and Frank, 2006). This configuration leads to a “winner-take-all” dynamic that generates sparse output, which is an appealing property for an action selection network so that only one output action is generated. However, this model has been called into question as electrophysiological studies have detected only weak functional connectivity between SPN, which is inconsistent with strong collateral inhibition needed for winner-take-all dynamics (Czubayko and Plenz, 2002; Tunstall et al., 2002; Koos et al., 2004). These SPN collaterals may thus be more important for other dynamical aspects of processing such as spike timing (Plenz, 2003) or the formation ensembles of SPN with coherent activity (Ponzi and Wickens, 2010).

**Figure 3:**
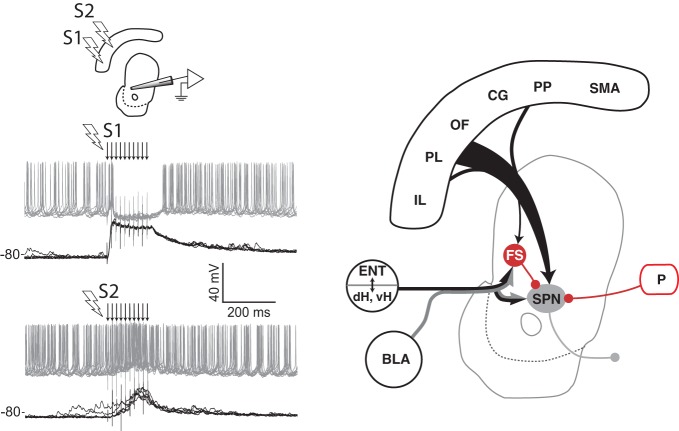
**Spatial sensitivity of striatal inhibition.** Intracellular recording (left panel) from one VS spiny projection neuron (SPN) in an anesthetized rat showing overlaid responses to tetanic electrical stimulation (arrows) in two different regions (S1, S2) of medial prefrontal cortex. Current injection into the neuron produces tonic firing (gray traces), which is inhibited by stimulation in one site (S1) and enhanced by stimulation in another (S2). The latency of the inhibitory component, its reversal potential (not shown), and data from other studies (see text) indicate that feed-forward inhibition from fast spiking (FS) striatal interneurons in VS and DMS is a likely source of this inhibition (right panel). Abbreviations are the same as for Figure 1. Adapted from Gruber et al. (2009b).

Although the functional role of SPN collaterals remains unclear, GABA-mediated inhibition within the striatum is an important component that shapes the response of SPN to afferent input (Rebec and Curtis, 1988; Mallet et al., 2005; Gruber et al., 2009b). Electrical stimulation of cortical afferents evokes an initial excitatory component followed by an inhibitory component on firing in the DMS, and infusion of GABA antagonists decreases the inhibition (Mallet et al., 2005; Galinanes et al., 2011). Furthermore, the inhibitory component depends on the spatial location of the stimulation in prefrontal cortex (Figure 3), a property essential for competition between loops linking cortex and striatum. A likely mechanism involved in these phenomena is feed-forward inhibition mediated by the GABAergic striatal fast spiking (FS) interneurons. Although these neurons comprise only a small proportion of striatal cells (Kawaguchi, 1993), they evoke large somatic inhibitory currents that inhibit SPN firing (Koos and Tepper, 1999). FS interneurons receive input from cortex, hippocampus, amygdala, and thalamus (Pennartz and Kitai, 1991; Kita, 1993; Bennett and Bolam, 1994), indicating that multiple afferents may be able to drive inhibition. FS interneurons in dorsal or VS phasically activate during tasks (Berke, 2008; Lansink et al., 2010), and suppressing their excitability in DLS causes dystonia-like effects (Gittis et al., 2011), suggesting that FS neurons are functionally important. FS and other GABAergic interneuron types targeting SPN are found throughout the striatum and may provide similar feed-forward inhibition across functional processing domains (Tepper, 2010). Electrophysiological recordings during spatial navigation for rewards have revealed that FS neurons exhibit coordinated activity in VS (Lansink et al., 2010), but exhibit uncoordinated activity in dorsal striatum (Berke, 2008). However, transient coordination among subsets of these neurons in dorsal striatum during operant task performance has been reported (Gage et al., 2010). Thus, the spatial scale of inhibition may vary across the striatum, and may depend on behavior. It remains to be determined whether such inhibition operates between nearby or distal striatal circuits. Other routes of inhibition within the basal ganglia are also likely to influence interactions among circuits. Extensive collaterals among GABAergic projections neurons appears to be a general characteristic found throughout the basal ganglia, including the output neurons in pallidum and substantia nigra (Millhouse, 1986; Parent et al., 2000), as well as GABAergic neurons of globus pallidus that project back to the striatum (Kita and Kitai, 1994; Bevan et al., 1998). Unlike the striatum, these pallidal collaterals have a robust inhibitory influence on neighboring neurons (Sadek et al., 2007; Sims et al., 2008). The convergent nature of both afferents to the striatum (McGeorge and Faull, 1989; Kincaid et al., 1998) and projections within the basal ganglia (Oorschot, 1996) place inhibitory mechanisms in these nuclei in a strategic position for mediating competition among parallel control systems.

### Striatal output: dopamine modulation of direct and indirect pathways

Competition for control of voluntary actions can also involve inhibition by the output of the basal ganglia on afferent structures. Two primary pathways through the basal ganglia have been identified with opposing effects on target neurons (Smith et al., 1998). The architecture and electrophysiology of the basal ganglia have led to a model of striatal function in which SPN projecting through the “direct” pathway have a disinhibitory effect on targets and promote action output, while those involved in the “indirect” pathway increase inhibition on target neurons and suppress action (Chevalier and Deniau, 1990; Mink, 1996). Thus, any input that can bias processing toward one pathway or the other could influence behavioral responding. One such input is dopamine. SPN involved in the direct pathway predominantly express D1 type dopamine receptors, whereas indirect pathway SPN express D2 type receptors (Gerfen et al., 1990; Surmeier et al., 1996). The proposed functional roles of the direct/indirect pathway and the select expression of dopamine receptors has been recently confirmed with optical stimulation of D1 or D2 expressing SPN in the DLS to evoke or inhibit locomotion, respectively (Kravitz et al., 2010). The level of dopamine is able to bias the relative excitability of the direct and indirect pathways by virtue of the opposing effects of D1 and D2 receptors on SPN excitability; the direct pathway is more excitable in high dopamine concentrations via D1-mediated excitation, whereas the indirect pathway is more excitable in low dopamine conditions via reduced D2-mediated suppression of SPN excitability (Albin et al., 1989; Surmeier et al., 2010).

Diffuse projections from brainstem dopamine-releasing neurons to cortical and limbic circuits include dense innervation of the striatum (Beckstead et al., 1979; Joel and Weiner, 2000). Many dopamine neurons initially respond to primary rewards by generating brief phasic increases of firing over tonic baseline, but this phasic response transfers to the earliest conditioned stimulus that predicts the delivery of reward as training progresses (Schultz, 1998; Pan et al., 2005). Omissions of expected rewards cause a depression of the tonic firing (Schultz, 1998). Dopamine neurons have thus been proposed (Montague et al., 1996) to signal errors in predicted rewards analogous to the learning signal in reinforcement learning theory that is essential for learning values of actions or future states (Sutton and Barto, 1998). Consistent with this hypothesis, dopamine neurons encode differences between expected and received reward in primates (Bayer and Glimcher, 2005), and dopamine has been shown to modulate plasticity at cortico-striatal synapses (Reynolds et al., 2001; Pawlak and Kerr, 2008; Shen et al., 2008). The direct and indirect pathways appear to have inverse learning rules wherein the D1 receptors in the direct pathway mediate post-synaptic long-term potentiation (LTP) following increases in dopamine and long-term depression (LTD) following decreases, while the opposite is true for D2 receptors (Shen et al., 2008). This suggests that the action-promoting direct pathway is strengthened following larger-than-expected rewards, whereas the action-suppressing D2 pathway is strengthened following smaller-than-expected rewards. This process has been confirmed in mouse DMS using optogenetic techniques to activate either D1 receptors, which promoted subsequent behavioral responding for light self-administration, or D2 receptors, which suppressed subsequent responding (Kravitz et al., 2012). In addition to biasing transmission between direct/indirect pathways and influencing plasticity, dopamine levels have also been proposed to bias the sensitivity of SPN to afferents from different sources such that cortical inputs dominate VS activity in conditions of low dopamine, while limbic inputs (amygdala and ventral hippocampus) dominate in conditions of high dopamine (Floresco et al., 2001; Goto and Grace, 2005). This is speculated to allow cortex to briefly exert control over behavior when outcomes are worse than expected so as to alter subsequent responding. This mechanism is consistent with data from multiple labs showing that manipulations of frontal cortex mostly impair the initial adaptation of rats following changes in reward contingencies such that responses become unexpectedly unrewarded (Birrell and Brown, 2000; Schoenbaum et al., 2002; Goto and Grace, 2005). However, this mechanism in VS does not preclude a role for hippocampus in flexible responding following disappointing outcomes, which could be mediated via cortex or directly in the striatum via diminished hippocampal input. Further data are needed to resolve this issue.

Dopamine neurons continue to respond to conditioned stimuli even in well-learned tasks (Schultz et al., 1993; Pan et al., 2005), and striatal dopamine levels encode relative value associated with stimuli (Gan et al., 2010). These phasic dopamine responses could thus be important for Pavlovian effects on behavior such as autoshaping and PIT, even in well-learned tasks. Consistent with this hypothesis, dopamine depletion in VS reduces engagement of operant responding (Nicola, 2010), whereas dopamine in both VS and DLS are involved in increased vigor via PIT (Hall et al., 2001; Corbit and Janak, 2007; Corbit and Balleine, 2011). The fact that PIT depends on both territories could be reflective of the spiral architecture in which VS neurons project to dopamine neurons in the substantia nigra (Groenewegen et al., 1993) that project to dorsal striatum (Beckstead et al., 1979; Joel and Weiner, 2000). Furthermore, the involvement of basolateral amygdala in Pavlovian effects such as PIT (Holland and Gallagher, 2003) and autoshaping (Parkinson et al., 2000a) may come from the ability of this input to activate SPN (O'Donnell and Grace, 1995) or to increase VS dopamine (Howland et al., 2002; Jones et al., 2010). Indeed, mice will operantly respond for optogenetic activation of amygdala input to VS through a mechanism that requires D1 receptors in the striatum, while optogenetic suppression of this input suppresses cue-evoked sucrose intake (Stuber et al., 2011). This latter finding is consistent with an ongoing role of dopamine in regulating behavior after acquisition. Interestingly, mice do not acquire analogous self-stimulation of cortical afferents to VS, suggesting that information from these different afferents have different impact in the striatum (Stuber et al., 2011).

These data indicate that the striatum is a nexus for multiple afferents involved in behavioral control, and has mechanisms needed for both competition and coordination among afferents to suppress or engage responding. These mechanisms involve inhibition and neuromodulatory control by dopamine for learning and on-line control of responding.

## Synthesis and computational perspectives

### Dissociated striatal circuits for triaging responses and generating actions by model-based or model-free control systems

Control systems involving different territories of the striatum have some distinct properties that are advantageous in particular tasks. Repeated reinforcement of stimulus driven responses develops S-R associations with sufficient strength so as to control behavior in tasks where such responding provides an adequate solution strategy (Tolman et al., 1946; Devan et al., 2011). The behavioral studies reviewed here indicate that these S-R mediated actions depend on DLS processing, are egocentric, and are insensitive to devaluation procedures. With repeated exposure, these S-R mediated actions can become habits (Barnes et al., 2005). Computationally, S-R control can be learned using “cashed” values of rewards acquired over many repetitions with simple algorithms (e.g., temporal difference) that do not build explicit models of environmental contingencies, and are thus considered to be “model-free” (Daw et al., 2005). Nonetheless, such algorithms can solve complex real-world problems, and can be executed quickly. They are, however, bound to particular stimuli and do not represent changes in environment until outcomes have been experienced repeatedly so as to weaken the S-R associations. Such control is therefore slow to adapt to changing environmental contingencies and is not intrinsically sensitive to motivational state because the reinforcement is not inferred prior to action.

The DMS is involved in flexible responding using hippocampal-dependent allocentric navigation, switching among cue dimensions for discriminations, and anticipating outcomes (Ragozzino et al., 2002; Yin et al., 2005; McDonald et al., 2008a). The DMS can therefore be characterized as associating a combination of stimuli, spatial contexts, responses, and reward outcomes. Computationally, formation of such antecedent-outcome associations necessitates representation of environmental contingencies, and is therefore considered to be a “model-based” learning system (Daw et al., 2005). Importantly, such systems allow mental testing of hypotheses about the outcomes of potential responses prior to action selection. Responding is thus sensitive to motivational state (e.g., devaluation) because the desirability of the reinforcement outcome can be inferred prior to the action. This type of control is therefore more flexible than the habit system.

**Figure 4:**
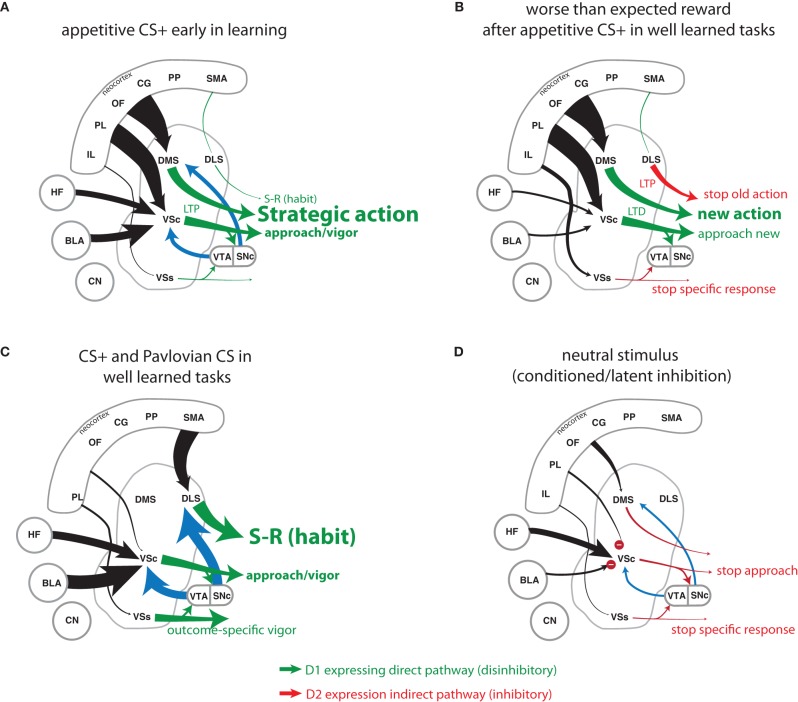
**Simple conceptual model of cortico-limbic processing in different scenarios. (A)** Presentation of a conditioned stimulus (CS+) early in learning evokes activity in amygdala, thereby triggering VSc activity and elevated dopamine (blue arrows) to engage approach and invigorate strategic actions in DMS. Following reward, direct pathway circuits undergo LTP via dopamine effects on D1 receptors. Many repetitions of such responding will eventually lead to habitual responding in tasks that can be solved by this response strategy. **(B)** Dopamine levels drop when expected rewards fail to occur, thus increasing sensitivity of striatal SPN to cortical input so as to alter response strategy. LTP ensues in the associated indirect DLS pathway to start the process of conditioned inhibition for that response, while LTD occurs on the associated DLS, DMS, and VS circuits that engaged the unrewarded action. These processes increase the likelihood of selecting a new action in the future. **(C)** Presentation of a Pavlovian CS engages a PIT mechanism involving additional activation of BLA, VSs, and dopamine to potentiate the associated S-R response in DLS as well as the general vigor via VSc. **(D)** Presentation of a CS with no associated reward in an excitatory context causes hippocampus to activate inhibition in the VSc so as to prevent orientation and task engagement. Furthermore, if the animal had previously responded to the CS- and received less reward than expected, LTP in the inhibitory indirect pathway would actively suppress these specific responses. Abbreviations are the same as for Figure 1, except that connectivity within the hippocampal formation (HF) is not illustrated.

Like the DMS, the VS and associated structures also form Pavlovian associations between stimuli, contexts, and affective outcomes. Unlike the DMS, however, evidence reviewed here indicates that its effect on motoric output is related to (i) approach behaviors needed to engage in operant responding and (ii) the vigor of responding (e.g., PIT), rather than selecting specific operant responses. We propose that the emotional memory network formed by VS, amygdala, ventral hippocampus, prefrontal cortex, and dopamine input can be conceptualized as triaging responses to stimuli based on their associated affective value in a context. When stimuli are associated with aversive outcomes, this system engages freezing and autonomic responses (e.g., bradycardia) via brainstem targets dissociated from habit or goal directed systems. For stimuli with positive valence, we propose the following simple model (Figure 4). Early in learning, hippocampal-amygdala circuits rapidly acquire response to a conditioned stimulus predictive of reward (CS+), and their output activates VS core and dopamine neurons so as to promote orientation and place preference, and invigorate non-specific activity so that the goal-oriented system can acquire contingencies and discover appropriate operant responses (Figure 4A). Later in learning, the CS+ activates amygdala, hippocampus, and neocortex, resulting in activation of VS-projecting dopamine neurons. Elevated dopamine in the VS biases SPN to hippocampal input and promotes direct pathway output to orient the animal and to trigger activity of dopamine neurons projecting to dorsal striatum. Elevated dopamine in the dorsal striatum promotes activation of the direct pathway to invigorate actions mediated by a competition between S-R systems involving the DLS and goal-oriented systems in the DMS. If the subsequent reward outcome is better than expected, a second bout of elevated dopamine causes LTP in the direct pathway via D1 receptors and LTD in the indirect pathway via D2 receptors, thereby increasing the likelihood of repeating the response. If the outcome is lower than expected, then the direct pathway depresses and the indirect pathway potentiates so that the response is more likely to be withheld following future stimulus presentations. Furthermore, reduced dopamine following negative reward prediction errors engages cortical control over striatal function so as to implement new response strategies (Figure 4B). Consistent with this simple model, damage, or inactivation of medial prefrontal or orbitofrontal cortex induces behavioral deficits that are most apparent following switches in task rules or outcomes (Ragozzino et al., 1999b; Birrell and Brown, 2000; Schoenbaum et al., 2002), and damage to DMS similarly impairs response flexibility (Devan et al., 1999; Ragozzino et al., 2002; Yin et al., 2005). Presentation of a Pavlovian CS during a well learned task evokes larger amygdala activations, promoting approach and by increasing VS core activity, and increasing vigor by increasing dopamine levels (Figure 4C).

### Aligning reinforcement learning with brain function: dopamine, inhibition, and context

Dopamine in the striatum is critical for learning and executing actions (Yun et al., 2004; Witten et al., 2011). The unique signaling properties of dopamine neurons and their striking similarity to reward prediction error signals have inspired proposals that the brain may use a form of reinforcement learning (RL) to make economic decisions. In RL, the reward prediction error signal is used to learn the value of action outcomes, and the decision-maker then biases selection of actions toward those with high value. Current models of cortico-striatal function developed under this elegant theory can replicate key features of flexible decisions in some economic choice tasks (Samejima et al., 2005; Daw et al., 2006; Glascher et al., 2010). However, the standard form does not perform well in other conditions (Niv et al., 2007; Zilli and Hasselmo, 2008; Collins and Frank, 2012). The addition of episodic or working-memory can improve RL model performance on tasks with delays or history dependence (Zilli and Hasselmo, 2008; Collins and Frank, 2012). Another candidate mechanism that may increase coherence of RL with biological function is the modulatory effects of emotion on vigor that may involve dopamine (Niv et al., 2007). The neuromodulatory effects of dopamine have been proposed to selectively enhance encoding of salient information (Berridge and Robinson, 1998; Gruber et al., 2004, 2006), increase response effort (Niv et al., 2007; Salamone et al., 2007), and bias the willingness to explore options (Parush et al., 2011; Humphries et al., 2012). Dopamine is involved in both the general and outcome-specific forms of PIT, which dissociate among striatal regions as described in a previous section. Furthermore, the representation of value may partially dissociate in striatum. Neurons in DMS can encode changing outcome values more quickly than in VS (Ito and Doya, 2009; Kimchi and Laubach, 2009), and animals are sensitive to reward value following VS damage (Balleine and Killcross, 1994; De Borchgrave et al., 2002). These data suggest parallel circuits for computing value in which the VS engages and invigorates non-specific actions based on general affective value (amount of food in this environment), whereas the DMS is involved in selecting actions based on specific expected outcomes of responses (left lever results in grape after a delay). As discussed by others (Pennartz et al., 2011), these features of the rodent striatum conflict with the current mapping of the standard actor-critic architecture of RL onto striatal circuits wherein the VS “critic” signals outcome value to dorsal striatal “actor” for action selection.

Dopamine neurons generate a variety of responses to stimuli associated with appetitive as well as aversive outcomes in rodents and primates (Mantz et al., 1989; Schultz et al., 1993; Pan et al., 2005; Brischoux et al., 2009; Matsumoto and Hikosaka, 2009). Measurement of dopamine release following such stimuli has revealed site specific effects; dopamine is elevated in VS shell following unconditioned rewarding stimuli (Bassareo et al., 2002; Aragona et al., 2009) and is elevated to a larger extent in frontal cortex than striatum following unconditioned aversive stimuli (Abercrombie et al., 1989; Bassareo et al., 2002). Furthermore, alteration of synaptic plasticity is selective to dopamine neurons projecting to frontal cortex and VS shell following aversive events, and selective to dopamine neurons projecting to VS core and shell following appetitive events (Lammel et al., 2011). However, *conditioned* stimuli associated with aversive or appetitive outcomes evoke dopamine release in VS core (Young, 2004; Gan et al., 2010). These data indicate functional heterogeneity of dopamine neurons projecting to different sites, and are consistent with the proposal (Bromberg-Martin et al., 2010) of distinct dopamine circuits mediating: (i) orientation and motivation, (ii) value learning, and (iii) detection of important sensory events. The two former functions map nicely onto the triaging function of the VS and strategic/flexible function of the DMS proposed here. The responses of dopamine neurons to aversive stimuli may fall under the latter category, and evidence suggesting a predominant cortical effect suggest that it may be useful for engaging cortex-driven alteration of responding to avoid negative affective states. Further investigation is needed to determine if elevated dopamine evoked by aversive stimuli could increase attention to relevant stimuli or affect decision-making without engaging approach.

Another biological component missing from the standard RL model is the learned suppression of responding through conditioned and latent inhibition. These could be mediated by multiple mechanisms in the striatum. For conditioned inhibition, worse-than-expected rewards potentiate the indirect pathway via D2 receptors so as to inhibit future responses. This is separate from LTD on the direct pathway. This has been shown with optogenetic stimulation of the indirect pathway (Kravitz et al., 2012), and is consistent with data from medicated Parkinson's patients who do not have a mechanism for brief reductions in dopamine and are impaired on learning from negative, but not positive, reward prediction errors (Frank et al., 2004). Another important mechanism for response suppression is the triaging function of the emotional system, which is important for guiding behavior to non-positive CS (Figure 4D). For neutral CS, this system closes the sensory-motor gate by means of latent or conditioned inhibition involving hippocampus and VS (McDonald et al., 2002, 2006). The emotional memory system may take advantage of the rapid learning capabilities, contextual sensitivity, and large memory capacity of hippocampal episodic memory to prevent responding to stimuli previously unrelated to reinforcement. We predict that this triaging would be particularly advantageous for focusing responding on productive actions in stimulus-rich environments that allow many possible unproductive actions. It could also unburden the goal-directed and habit systems from learning associations for low-valued outcomes that could interfere with encoding of high-value associations. On the other hand, such gating out of sensorimotor responding by incidental learning in hippocampus and amygdala can impair behavioral flexibility when previously neutral stimuli become predictive of rewards.

The intermediate and ventral portions of hippocampus and associated structures innervate both the DMS and VS, and are thought to provide context. Although context is often associated with space, it can also represent temporal ordering in spatial and non-spatial domains (Fortin et al., 2002; Howland et al., 2008), segment phase during spatial alternation (Wood et al., 2000), configuration of stimuli (Rudy and Sutherland, 1989), task rules (Satvat et al., 2011), and internal drive states (Kennedy and Shapiro, 2004). Thus, the ability of hippocampus to pre-play task-related information prior to choices, as has been identified in dorsal hippocampus (Johnson and Redish, 2007; Ferbinteanu et al., 2011), could impart a great deal of information to DMS and frontal cortex in support of flexible decisions. This input is expected to be particularly important for decision making in conditions of high spatial or contextual ambiguity, or when complex configurations of stimuli or their temporal ordering cue appropriate behavior. The ability of hippocampus to separate patterns by generating orthogonal representations (O'Reilly and Rudy, 2001) may additionally facilitate learning about salient features of complex input that may otherwise be generalized in amygdala or cortico-striatal circuits. Conversely, ventral hippocampus is involved in contextual gating of activity to unrewarding stimuli. For instance, reversal learning is slower in familiar contexts as compared to novel contexts in rats with intact hippocampus, but this slowing is eliminated by lesions of ventral hippocampus (McDonald et al., 2002, 2006). This lesion-induced enhancement of reversal learning reflects removal of a context-specific inhibition, possibly by suppressing flexible approach to neutral stimuli via the VS. These lesions also enhance CPP, again reflecting a context-specific inhibitory influence possibly mediated by blocking amygdala access to VS (McDonald and White, 1993, 1995b). These inhibitory functions are separate but not exclusive of the proposed excitatory hippocampal gating functions mediated by glutamatergic input to SPN conjunctive with other afferents (O'Donnell and Grace, 1995).

One potential mechanism for the inhibitory hippocampal gating function is competition for striatal control mediated by inhibitory mechanisms in the striatum. This inhibition may also play a role in the competition between DMS-mediated “model-based” control and DLS-mediated “model-free” control observed in spatial navigation tasks (McDonald and White, 1994; Devan et al., 1999; Thorn et al., 2010), impulse suppression (Winstanley et al., 2006), and the hypothesized suppression of extinction functions of the ventral networks by DMS during reversal learning paradigms (McDonald et al., 2008b). However, these systems do not always compete. Tasks requiring integration of the unique processing capabilities of multiple systems leads to synergistic interactions (White and McDonald, 2002).

### Pathology of decision-making systems

The processing in networks among cortex, basal ganglia, hippocampus, amygdala, hypothalamus, and brainstem neuromodulatory systems forms a distributed control system with multiple learning systems that operate in parallel for controlling behavior. This distributed architecture has an advantage of containing few single points of *total* failure; much of the work discussed in this review shows that rats can learn to perform many tasks, albeit sub-optimally, following damage to any one striatal region or its cortico-limbic afferents. However, it does provide multiple points for *partial* failure in that damage to one of many structures can lead to the same deficit, and dysfunction of one control system can alter processing in others. These features obfuscate the etiology of decision-making deficits accompanying many mental illnesses. For example, schizophrenia among other illnesses is associated with complex symptomatology including impairments in working-memory, response perseveration, and outcome valuations as well as sensorimotor gating (Braff and Geyer, 1990; Gold et al., 2008). How much of the cognitive effects derive from dysfunction of the proposed limbic triaging system remain to be determined (Grace, 2000; Bast, 2011). Furthermore, the limbic system is involved in or affected by brain systems mediating stress (Herman et al., 2005), mood (Price and Drevets, 2012), and addictions (Everitt et al., 1999), suggesting that it is a conduit by which these affective states can influence decisions in healthy and ill brains.

## Conclusion

We propose that a ventral emotional network including amygdala and ventral portions of hippocampus, striatum, and medial prefrontal cortex performs triaging of responding based on affective associations so as to avoid unpleasurable stimuli, ignore inconsequential stimuli, and approach pleasurable stimuli. The approach and accompanying gating of sensory signals promote the selection of operant actions by model-based or model-free systems involving dorsal striatum. The rapid learning rate and large capacity for S-O (amygdala) and sensory-sensory (hippocampus) associations may provide a solution for the problem of learning to thrive in large environments that present many unproductive stimuli and permit many inconsequential actions, which is computationally difficult to solve (Sutton and Barto, 1998). The triaging system may allow animals to identify, following little experience, hazards to avoid and opportunities to exploit for benefit. This alone may facilitate survival by providing simple outcome-predictive control over elementary behaviors such as foraging. It may further aid more complex behaviors by unburdening model-based and model-free control from processing or remembering irrelevant information. In addition to gating responses, this ventral system also appears to invigorate actions as well as provide rich contextual information that aids the model-based control system in complex discriminations and learning. Potential downsides of this triaging system are poor decision-making under states of heightened emotion, and slow adaptation when affective associations of stimuli change qualitatively (e.g., neutral to positive). However, we suggest that slow adaptation of this system may aid learning in some appetitive tasks following contingency changes by keeping animals engaged in tasks while new response strategies can be learned, despite temporary reduction in rewarded responding.

Learning and memory systems involved in behavioral control appear to function in parallel, even when they are not part of the dominant response strategy. However, these non-dominant associations may become relevant when adapting to changing environmental contingencies. For reversal learning of a habit, suppression of both the dominant response and latent/conditioned inhibition to previously-irrelevant stimuli must accompany formation of new associations between stimuli, responses, and values. Dysfunction of any of these processes will retard reversals. Thus, a multiple-systems-level conceptualization may help drive new insight to decision-making processes in the brain, particularly regarding the role of emotional memories and the control of habitual responding, and may come to illuminate the processes underlying many examples of inflexible or irrational human behavior involving emotion (Ditto et al., 2006; Van Den Bos et al., 2008).

### Conflict of interest statement

The authors declare that the research was conducted in the absence of any commercial or financial relationships that could be construed as a potential conflict of interest.

## Abbreviations

CPP: conditioned place preference
CS: conditioned stimulus
DLS: dorsolateral striatum
DMS: dorsomedial striatum
PIT: Pavlovian-to-instrumental transfer
RL: reinforcement learning
R-O: response-outcome
S-O: stimulus-outcome
S-R: sensory-response
VS: ventral striatum (a.k.a. nucleus accumbens).
